# Alkali-Modified Biochar Derived from Waste Bamboo Powder for the Effective Adsorption of Perfluorooctanoic Acid

**DOI:** 10.3390/molecules31030568

**Published:** 2026-02-06

**Authors:** Long Lu, Hongbin Wang, Junfeng Zhao, Mei Zhang, Xuying Zheng, Dapeng Luo, Yongliang Sun, Jinyan Yang

**Affiliations:** 1 School of Architecture and Civil Engineering, Chengdu University, Chengdu 610106, China; lulong@stu.cdu.edu.cn (L.L.);; 2 Sichuan Environmental Protection Key Laboratory of Persistent Pollutant Wastewater Treatment, Sichuan Normal University, Chengdu 610066, China; 3 Sichuan Academy of Eco-Environmental Sciences, Chengdu 610041, China; 4 College of Architecture and Environment, Sichuan University, Chengdu 610227, China

**Keywords:** waste bamboo powder, KOH modification, perfluorooctanoic acid, adsorption performance

## Abstract

Bamboo powder waste generated from bamboo processing serves as an ideal feedstock for biochar (BC). This study employed potassium hydroxide (KOH) to modify biochar derived from bamboo powder waste, activating it at different temperatures (700 °C, 800 °C, and 900 °C) to yield samples designated KBC-700, KBC-800, and KBC-900, respectively. The physicochemical properties and pore structures of the modified biochar were characterized using SEM, specific surface area and pore size analysis, FT-IR, Raman spectroscopy, XRD, and zeta potential measurements. The adsorption performance of the modified biochar toward PFOA was investigated using kinetic and thermodynamic models, examining the effects of the solution pH, adsorbent dosage, and temperature. Results indicate that KBC-900 exhibits a significantly enhanced specific surface area (up to 2924.7 m^2^ g^−1^), reduced surface oxygen-containing functional groups, increased carbon skeleton aromatization, and expanded mesoporous channels. Under initial conditions of pH = 3 and reaction temperature of 298 K, KBC-900 achieved a PFOA adsorption capacity of 366.7 mg g^−1^ with a removal efficiency of 91.67%. The adsorption process conformed to pseudo-first-order and pseudo-second-order kinetic models as well as the Freundlich model. The adsorption equilibrium was reached within 12 h, indicating multi-layer adsorption dominated by chemisorption on a heterogeneous surface. Thermodynamic parameters indicate the adsorption reaction is an exothermic process. After five cycles of regeneration, KBC-900 maintained a removal efficiency of 75.69%. This study provides an efficient and reliable solution for removing PFOA from water.

## 1. Introduction

**Figure 1:**
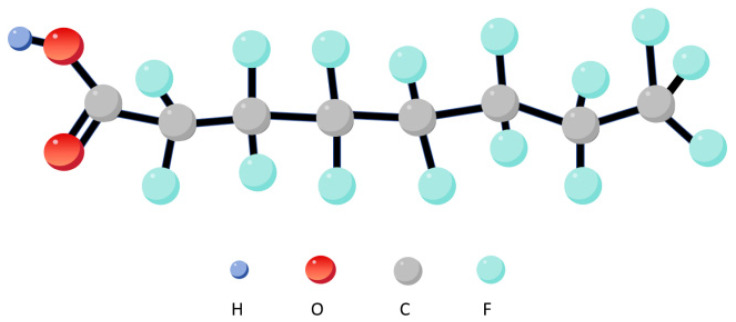
Three-dimensional structure diagram of PFOA.

Per- and polyfluoroalkyl substances (PFAS) are a class of stable, widely used synthetic organic compounds [1]. Recently, they have garnered global attention as persistent organic pollutants [2]. Perfluorooctanoic acid (PFOA), a representative perfluorinated compound, is featured with a molecular structure combining a hydrophobic perfluorocarbon chain with a hydrophilic carboxyl group, which is illustrated in Figure 1. This amphiphilic structure confers some water solubility [3], while the high C-F bond energy (approximately 485 kJ/mol) ensures exceptional stability [4]. These exceptional physicochemical properties render its widespread application in industrial production and consumer goods manufacturing, including fluorinated rubber, paints, fire-resistant foams, and leather products [5,6,7].

PFOA is difficult to degrade naturally in the environment, while it exhibits bioaccumulation and potential ecotoxicity [8]. In 2019, the United Nations Environment Programme listed PFOA and its salts under the Stockholm Convention to restrict their use [9]. China’s Ministry of Ecology and Environment also included PFOA substances in the Key Controlled New Pollutants List (2023 Edition). Although the production and use of PFOA have been restricted in some countries and regions, it is still frequently detected in many areas due to historical emissions and its persistence [10]. In 2015, PFOA concentrations detected in the Chaohu Lake basin were 33.3 ng L^−1^, while those in the Taihu Lake basin were 24.9 ng L^−1^ [11]. In 2020, PFOA concentrations detected in Bohai Bay were 14.96 ng L^−1^ [12], and 20.90 ng L^−1^ at the Pearl River Estuary in 2021 [13]. Although these concentrations remain below the national drinking water standard limit (80 ng L^−1^), their widespread detection and potential ecological health risks continue to attract significant attention [14]. Identifying efficient and cost-effective PFOA removal technologies has thus become one of the critical challenges requiring urgent solutions in the field of environmental engineering [15].

Traditional water treatment processes (such as coagulation, catalysis, and filtration) exhibit low removal efficiency for PFOA and incur high infrastructure costs. Based on a comprehensive review of domestic and international research on PFOA removal, applicable technologies include: advanced oxidation, photocatalytic degradation, bioremediation, adsorption methods, ultraviolet irradiation, and electrochemical methods [16,17,18,19]. Technologies like advanced oxidation and photocatalytic degradation may generate unpredictable intermediate products during treatment, posing risks of secondary pollution [20]. Bioremediation methods feature lengthy treatment cycles and reduced degradation efficiency [21]. In contrast, adsorption is considered one of the most promising technologies for removing PFOA from water due to its operational simplicity, low cost, and high removal efficiency [22,23]. Common adsorbents include activated carbon, resins, minerals, and biochar [24,25]. Among these, biochar—a low-cost, porous, carbon-rich material—offers excellent properties such as abundant raw material sources, good environmental compatibility, and a tunable specific surface area. It is widely used for removing heavy metals, pesticides, and other persistent organic pollutants from water [26,27]. The properties of biochar are influenced by multiple factors, particularly the raw material and pyrolysis temperature [28]. Murray et al. [29] prepared granular activated carbon and ultrafine powdered activated carbon from woody feedstocks, with the ultrafine powder demonstrating a 150% improvement in PFOA treatment efficiency. Lei et al. [30] found that increasing the pyrolysis temperature to 900 °C boosted the adsorption capacity of ordered mesoporous carbon for PFOA by 40%, a mechanism dependent on the synergistic effects of hydrophobic interactions and electrostatic attraction. Yu et al. [31] observed that bamboo biochar pyrolyzed at 500 °C exhibited more developed pore structures compared to those at 300 °C and 700 °C, achieving the highest PFOS removal efficiency (49.45%). Ge et al. [32] demonstrated that bamboo powder activated with KOH achieved a specific surface area of 562 m^2^ g^−1^ at 900 °C pyrolysis, with an MB removal rate of 99.5% and a maximum adsorption capacity of 67.71 mg g^−1^. Liu et al. [33] discovered that modified bamboo charcoal prepared by pyrolyzing KOH-mixed BB-900 at 900 °C significantly enhanced tetracycline degradation efficiency, achieving a removal rate of 91.1% within 60 min. Current research indicates that KOH chemical activation is an efficient biochar modification method. It constructs a developed micropore structure and high specific surface area on the carbon framework by leveraging the etching effect of KOH at high temperatures, thereby significantly increasing pollutant adsorption sites [34,35].

China is rich in abundant bamboo resources, ranking first globally in bamboo production [36]. As a rapidly growing and highly renewable biomass resource, bamboo is commonly utilized as a construction and decorative material. The waste bamboo powder generated from bamboo processing serves as an ideal raw material for biochar production [37]. In this study, biochar (BC) derived from waste bamboo powder was modified using KOH and activated at three temperatures—700 °C, 800 °C, and 900 °C—yielding modified biochar samples designated KBC-x (x = 700, 800, 900). The physicochemical properties were systematically analyzed using scanning electron microscopy (SEM), BET surface area and pore size analysis, Fourier transform infrared spectroscopy (FT-IR), Raman spectroscopy, X-ray diffraction (XRD), and zeta potential measurement. The adsorption performance, adsorption mechanism, regeneration capability, and influencing factors of the modified biochar toward PFOA were investigated. This study utilized waste bamboo powder as a precursor to preparing high-performance biochar through KOH modification. This approach not only aligns closely with China’s current national priorities in addressing emerging pollutants and advancing the “carbon peak and carbon neutrality” strategy, thereby achieving resource recycling, but also provides a novel material option for the efficient removal of PFOA from water.

## 2. Results and Discussion

### 2.1. Characterization of Biochar

#### 2.1.1. SEM

**Figure 2:**
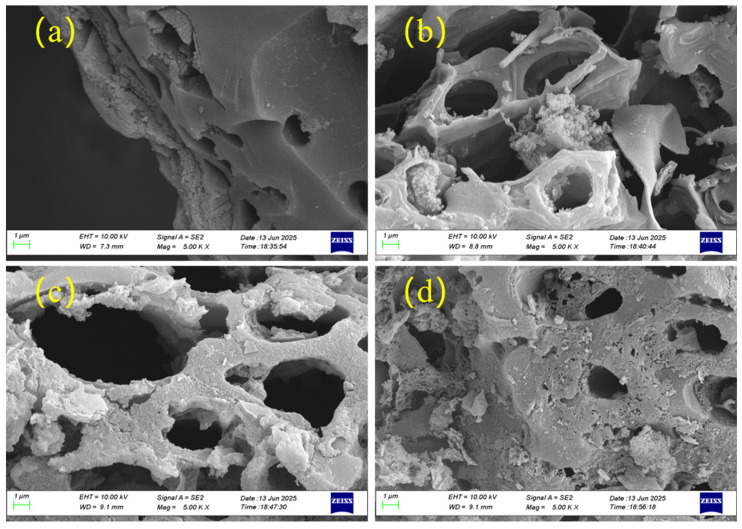
(**a**–**d**) show SEM images of BC (**a**), KB-700 (**b**), KBC-800 (**c**), and KBC-900 (**d**) at 5000× magnification, respectively.

Pyrolysis temperature is a key factor determining the microstructure of bamboo-based biochar. SEM images of BC, KBC-700, KBC-800, and KBC-900 are shown in Figure 2. Most biochar obtained from bamboo powder pyrolysis exhibits a relatively coarse surface, while the surface of BC (Figure 2a) appears relatively dense and smooth. SEM images of KBC-700, KBC-800, and KBC-900 (Figure 2b–d) reveal that after KOH activation, some biochar particles are disrupted with visible etching marks, exhibiting a significantly developed pore structure. Both the specific surface area and pore volume are substantially enhanced. As pyrolysis temperature increased, KOH etching intensified, leading to near-complete decomposition of surface particles, further refinement of the pore structure, and coarsening of surface morphology. These microstructural changes align with Ge’s research findings [32]. Specifically, KBC-700 (Figure 2b) developed larger pores with a relatively smooth surface, while KBC-800 (Figure 2c) exhibited markedly increased roughness. As for KBC-900 (Figure 2d), due to the complete decomposition of organic matter at high temperatures, its surface became rougher with a more porous and loose structure, revealing some nanoscale pores on the surface. These increased nanoscale pores may be closely related to PFOA adsorption.

#### 2.1.2. Specific Surface Area and Pore Structure

**Figure 3:**
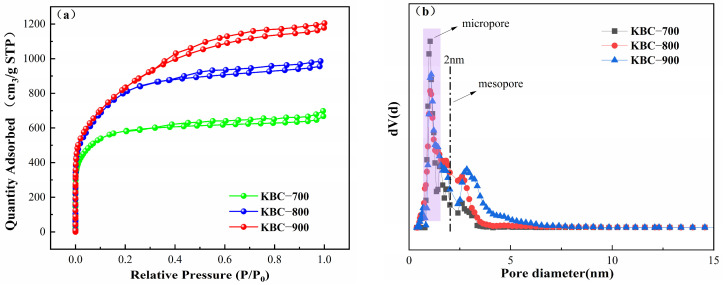
Adsorption–desorption isotherms (**a**) and pore size distribution (**b**) of the adsorption obtained at different temperatures.

According to the definition by the International Union of Pure and Applied Chemistry (IUPAC), pores with diameters less than 2 nm are termed micropores, pores with diameters between 2 and 50 nm are termed mesopores, and pores with diameters greater than 50 nm are termed macropores [38]. The N_2_ adsorption/desorption isotherms of biochar are shown in Figure 3a. According to IUPAC classification, the isotherms of all three biochar samples exhibit H4-type hysteresis loops, characteristic of activated biochar solids containing narrow fissure pores [39,40]. KBC-700 exhibits a relatively narrow hysteresis loop, while KBC-900 displays the broadest loop. Samples adsorb substantial nitrogen at low pressures, indicating the presence of larger mesopores or micropores. Figure 3b reveals that KOH activation transforms the material into a micro-mesoporous structure. As activation temperature increases, pore volume decreases in the 1–2 nm size range while increasing in the 2–5 nm range, indicating pore expansion at higher temperatures. These enlarged pores may provide adsorption sites for PFOA [41,42]. Model calculations for KBC-700, KBC-800, and KBC-900 are shown in Table 1. At a pyrolysis temperature of 900 °C, the specific surface area of KBC-900 significantly increased to 2924.7 m^2^ g^−1^, exceeding that of KBC-700 (2398.6 m^2^ g^−1^) and KBC-800 (2896.7 m^2^ g^−1^), indicating enhanced porosity development with rising pyrolysis temperature. The total pore volume also increased from 1.310 m^3^ g^−1^ at 700 °C to 1.5798 m^3^ g^−1^ at 900 °C. These results demonstrate that activation temperature significantly regulates the pore structure of the material, and high-temperature carbonization is an effective strategy for preparing high-porosity biochar.

#### 2.1.3. FTIR

**Figure 4:**
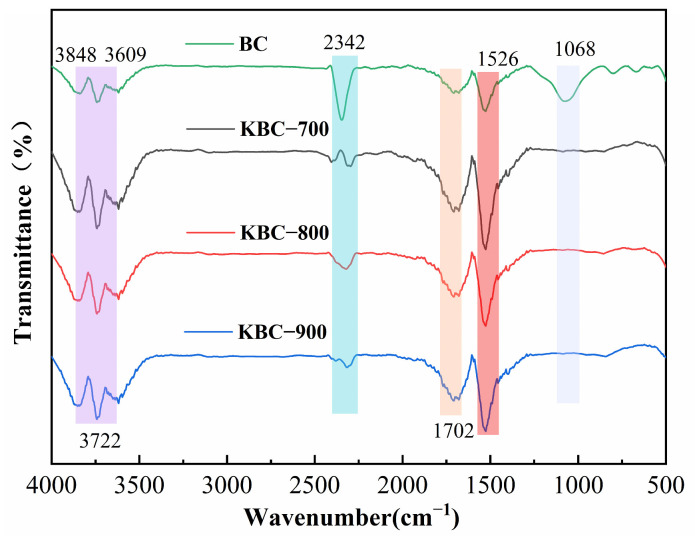
FT-IR spectra of BC, KBC-700, KBC-800, and KBC-900.

The surface chemistry of biochar is closely related to its adsorption capacity. The FT-IR spectra of BC and KBC-series bamboo biochar are shown in Figure 4. For all materials, the spectral band in the range of 3742–3609 cm^−1^ corresponds to the stretching vibration of surface O–H groups [43]. The prominent characteristic peak observed at 1068 cm^−1^ in BC is attributed to C-O stretching vibrations of ether, ester, and phenolic groups [44], indicating the presence of abundant oxygen-containing functional groups on the BC surface. However, this characteristic peak completely disappears in all modified samples, demonstrating that high-temperature KOH treatment effectively removed oxygen-containing functional groups. Simultaneously, all samples retained and enhanced the prominent aromatic ring C=C backbone vibration peak at 1526 cm^−1^, indicating that the aromatic structure of the carbon matrix was preserved and strengthened during modification [45]. The removal of oxygen-containing functional groups substantially increased the hydrophobicity of the biochar surface, facilitating hydrophobic interactions with the perfluoroalkyl chains of PFOA molecules [34,46]. Furthermore, the enhanced aromatic structure provided a foundation for π-π interactions between biochar and PFOA [47].

#### 2.1.4. Raman Spectroscopy

**Figure 5:**
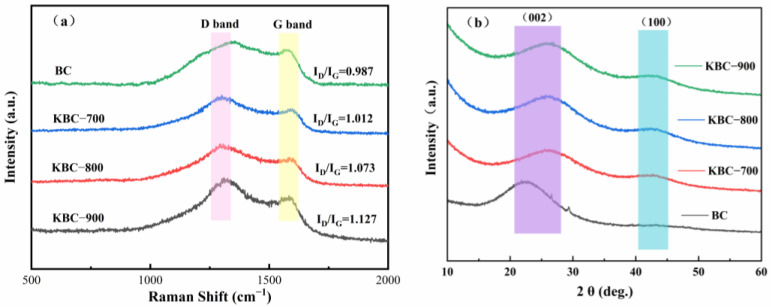
Raman spectra (**a**) and X-ray diffraction patterns (**b**) of BC, KBC-700, KBC-800, and KBC-900.

Raman spectroscopy analysis revealed the evolution patterns of the ordered structure and defect density in the biochar carbon skeleton (Figure 5a). Characteristic peaks were observed at 1350 cm^−1^ (D band, disordered structure/defects) and 1580 cm^−1^ (G band, sp^2^ carbon stretching vibration) for all samples. The intensity ratio of the D band to the G band (ID/IG) is commonly used to characterize the disorder or defect level in carbon materials [48]. The ID/IG value increased gradually from 0.987 in the raw BC to 1.012 in KBC-700, 1.073 in KBC-800, and peaked at 1.127 in KBC-900. This indicates that higher pyrolysis temperatures introduce more structural defects and disorder into the biochar carbon skeleton. Such structural evolution is typically accompanied by the development of nanoscale pore structures and an increase in edge sites. This not only significantly enhances the material’s specific surface area, providing more adsorption sites, but may also strengthen the van der Waals forces between the carbon skeleton and the hydrophobic fluorocarbon chains of PFOA [49]. This result aligns with KBC-900’s advantages in the specific surface area and pore structure, indicating that KOH high-temperature activation effectively promotes pore development while intensifying carbon layer structural disorder. This may contribute to its enhanced adsorption performance.

#### 2.1.5. XRD Analysis

The XRD patterns of biochar reveal the evolution of its crystal structure (Figure 5b). All samples exhibit a broad diffraction peak near 2θ = 24° corresponding to the (002) crystal plane, indicating that the prepared biochar primarily consists of disordered carbon structures [50]. Compared to BC, the (002) diffraction peak in the KBC-series samples shows a significant rightward shift and reduced intensity, reflecting decreased interlayer spacing, thinner stacking thickness of graphite microcrystals, and overall reduced order [51]. The (100) diffraction peak at 2θ ≈ 44° gradually became more pronounced in KBC samples while remaining nearly invisible in BC, indicating that KOH activation promoted the formation of in-plane ordered structures and increased edge sites. These structural changes align with the increasing ID/IG ratio observed in Raman spectroscopy. Collectively, they demonstrate that high-temperature KOH activation introduces structural defects and disorder while simultaneously promoting the development of nanoscale pores and the exposure of edge carbon structures. This facilitates an enhanced specific surface area and increased adsorption sites, providing the structural foundation for PFOA adsorption.

### 2.2. Effects of Biochar Dosage and pH Value

**Figure 6:**
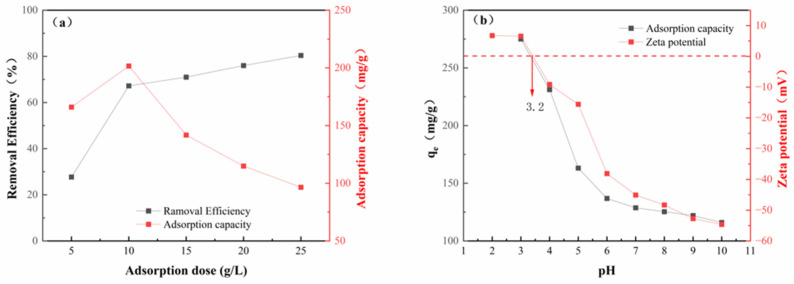
Effect of KBC-900 dosage on PFOA adsorption (**a**); effect of pH on PFOA adsorption and zeta potential (**b**).

The effect of adsorbent dosage on the removal efficiency and adsorption capacity of KBC-900 for PFOA is shown in Figure 6a. As the adsorbent dosage increased from 5 mg to 25 mg, the PFOA removal efficiency gradually rose, reaching a maximum of 80.4% at 25 mg. The adsorption capacity initially increased and then decreased within the 5–25 mg range, achieving its maximum value (201.6 mg g^−1^) at a dosage of 10 mg. This was primarily due to an increase in the total number of active sites. However, beyond a certain threshold, adsorption capacity began to decline. This may be related to the reduced utilization efficiency of adsorption sites at high-adsorbent concentrations. Under fixed total pollutant conditions, increasing the dosage reduces the solute-to-adsorbent ratio, preventing available sites from reaching saturation [52]. Simultaneously, particle agglomeration and site overlap further diminish mass transfer efficiency and the effective surface area per unit mass of adsorbent. Therefore, to maximize adsorption capacity utilization, an adsorbent dosage of 10 mg was selected as the optimal amount. Under these experimental conditions, a removal efficiency of 67.2% was achieved.

The effect of the initial solution pH on the adsorption capacity of KBC-900 for PFOA is shown in Figure 6b. It is evident that the solution pH significantly influences the adsorption of PFOA by KBC-900. The adsorption capacity reaches its optimum at pH = 3. As the pH gradually increases from acidic to neutral, the adsorption capacity decreases significantly. Subsequently, when the pH shifts to alkaline, the adsorption capacity declines slowly. The optimal adsorption pH of PFOA on materials is 3, consistent with the optimal pH range reported by most researchers [31,53,54]. The zeta potential of the adsorbent surface at different solution pH values is shown in Figure 6b. The zero-charge point was found to be around pH 3.2. Since the pKa value of PFOA is 2.8 [55], when the solution pH is below the zero-charge point of biochar, the biochar surface carries a positive charge. At this point, most PFOA in the solution exists as an anion. The positively charged biochar exhibits strong electrostatic attraction with the negatively charged PFOA anions, constituting the dominant mechanism for efficient adsorption [56]. This phenomenon of electrostatic attraction dominating adsorption under acidic conditions (high protonation of PFOA) is consistent with Zhou’s findings [42], which revealed that the protonated form of PFOA (HFPO-DA) at a low pH significantly inhibits its adsorption onto activated carbon. The fundamental reason lies in the fact that protonation weakens the electrostatic attraction between PFOA and positively charged adsorption sites. At solution pH values above the biochar’s zero-charge point, the biochar surface becomes negatively charged. Electrostatic repulsion between the biochar and PFOA anions gradually increases, leading to a significant decrease in adsorption capacity as the pH rises. In the neutral-to-alkaline range, the rate of adsorption capacity decline slows but continues to decrease gradually. At this point, adsorption primarily relies on weaker hydrophobic interactions [57].

### 2.3. Influence of Reaction Time on PFOA Adsorption

**Figure 7:**
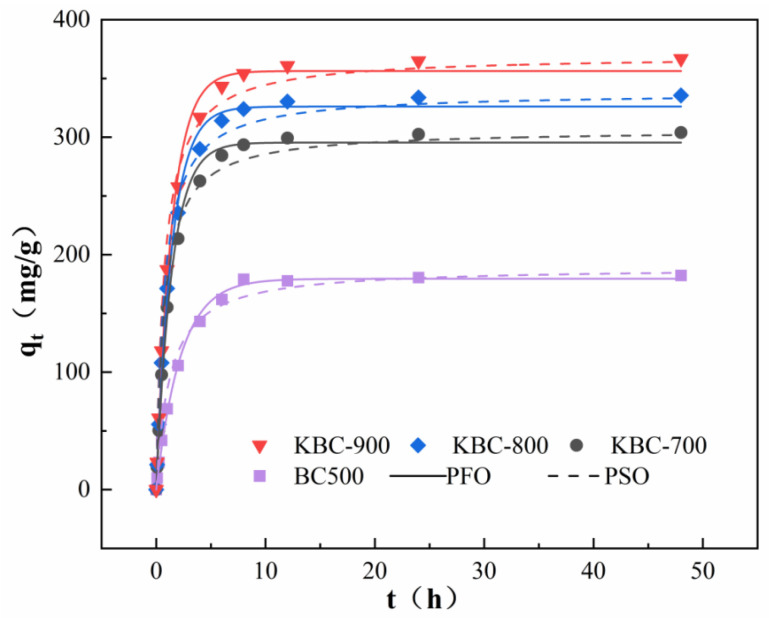
Adsorption kinetics of PFOA by BC and KBC-series biochar.

Figure 7 shows the results of fitting experimental data using pseudo-first-order and pseudo-second-order kinetic models: the adsorption equilibrium of PFOA on biochar is essentially achieved within 24 h. All samples exhibited rapid adsorption rates during the initial phase (approximately the first 10 h), with adsorption capacity increasing sharply. Subsequently, the rate of increase slowed, gradually approaching the adsorption equilibrium. It is clearly evident that the adsorption performance of the KBC-series modified biochar significantly outperforms that of unmodified biochar. Analysis of the fitting parameters (Table 2) and adsorption kinetics curves (Figure 7) indicates that the adsorption process was simultaneously well described by both pseudo-first-order and pseudo-second-order kinetic models, with the pseudo-second-order model exhibiting a higher fitting accuracy. The calculated equilibrium adsorption capacities (q_e_) are 305.74 mg g^−1^ (KBC-700), 336.35 mg g^−1^ (KBC-800), and 369.49 mg g^−1^ (KBC-900). These values are also closer to the experimentally measured equilibrium adsorption capacities of 304 mg g^−1^ (KBC-700), 335.5 mg g^−1^ (KBC-800), and 366.7 mg g^−1^ (KBC-900). This indicates that both physical and chemical adsorption occur during PFOA adsorption by modified biochar, with chemical adsorption being dominant. The kinetic curves reveal that all three materials exhibit an initial rapid increase in the adsorption rate followed by a gradual approach to the equilibrium. Among them, KBC-900 demonstrates the highest adsorption capacity and the fastest adsorption rate.

### 2.4. Influence of Initial Concentration on PFOA Adsorption

**Figure 8:**
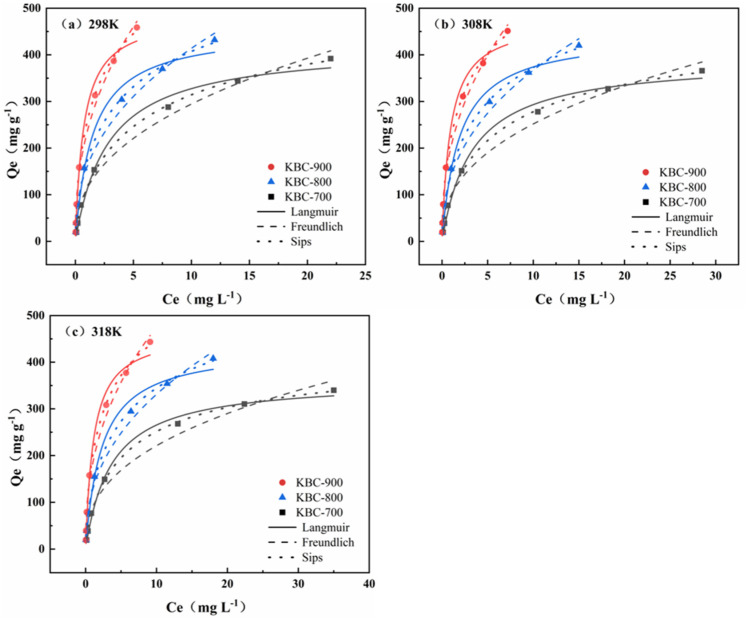
Three model fits for KBC-series biochar at different temperatures: 298 K (**a**), 308 K (**b**), 318 K (**c**).

Adsorption isotherms are key to evaluating adsorbent–adsorbate interactions [58]. Isothermal adsorption experiments were conducted at different temperatures, and Langmuir, Freundlich, and Sips adsorption isotherm models were employed to investigate the adsorption mechanism. As shown in the fitting results of the three models (Figure 8) and the fitting parameters (Table 3), the adsorption amount of PFOA decreases with increasing temperature, indicating that elevated temperatures are detrimental to adsorption. According to the Langmuir model fitting, the theoretical maximum adsorption capacity of PFOA on KBC-900 is 487.89 mg g^−1^ (at 298 K). The Freundlich model exhibits a higher correlation coefficient R^2^ than the Langmuir model, and all 1/n values are less than 0.5. This indicates preferential adsorption and suggests that the biochar surface possesses high heterogeneity, potentially involving multi-layer adsorption. Intermolecular interactions cannot be ignored [59]. The Sips model provides a better fit and can also describe the aforementioned adsorption behavior. The 1/β values fall between 0 and 1, further indicating that the adsorbent surface is heterogeneous and that the adsorption process involves multi-layer adsorption on a non-uniform surface [55]. Table 4 presents a comparative analysis of PFOA adsorption with other reported modified biochar materials. Compared to other modified biochar, the PFOA adsorption capacity of KBC-900 in this study (487.89 mg g^−1^) already outperforms most modified biochar materials. Its adsorption capacity is comparable to that of the material studied by Deng et al. [60] but with a shorter adsorption equilibrium time. Compared to other materials with shorter equilibrium times [61,62,63], KBC-900 exhibits a favorable balance between adsorption capacity, adsorbent dosage, and equilibrium time, demonstrating highly efficient and economical adsorption properties.

### 2.5. Thermodynamic Analysis

The calculation of adsorption energy is a critical step in investigating adsorption behavior and evaluating its thermodynamic properties [67]. Table 5 lists the calculated thermodynamic parameters for three biochar materials: KBC-700, KBC-800, and KBC-900. At different temperatures, ΔG_0_ is negative for all materials, indicating that their adsorption of PFOA is a thermodynamically spontaneous process. Furthermore, the absolute value of ΔG_0_ decreases with increasing temperature, reflecting that elevated temperatures reduce the adsorption driving force and hinder the reaction. The ΔG_0_ values for KBC-900 (−14.92 to 17.17 kJ/mol) are highly consistent with the spontaneity observed in the study by Wu et al. (ΔG_0_ = −13.83 to −20.22 kJ/mol) [68]. Meanwhile, the negative value of ΔH_0_ indicates that the adsorption process is an exothermic reaction. The range of negative ΔH_0_ values (−20.27 to −21.33 kJ/mol) closely aligns with the exothermic behavior of PFOA adsorption on activated carbon reported by Fab et al. (ΔH_0_ = −21.66 kJ/mol), further thermodynamically validating the inhibitory effect of elevated temperatures on PFOA adsorption [61]. On the other hand, the consistently negative ΔS_0_ values indicate a reduction in the system’s degrees of freedom during adsorption, consistent with the characteristic immobilization of molecules at the solid–liquid interface. These thermodynamic parameters further support the conclusion that adsorption is a spontaneous exothermic process [69].

### 2.6. Practical Application and Regenerative Performance

**Figure 9:**
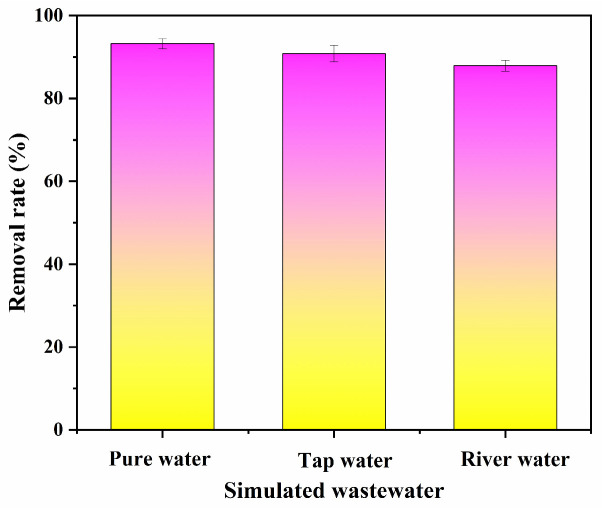
Adsorption of PFOA by KBC-900 in different water types.

The effect of simulated wastewater from different water sources on KBC-900’s adsorption of PFOA is shown in Figure 9. Among the three water types, river water exhibited the most significant impact, likely due to certain impurities present. Tap water also showed some effect, possibly because residual chlorine in tap water negatively affected PFOA adsorption. KBC-900 achieved removal rates exceeding 90% for simulated wastewater from all three water sources. These results demonstrate that KBC-900 is suitable for adsorbing PFOA from various water bodies.

**Figure 10:**
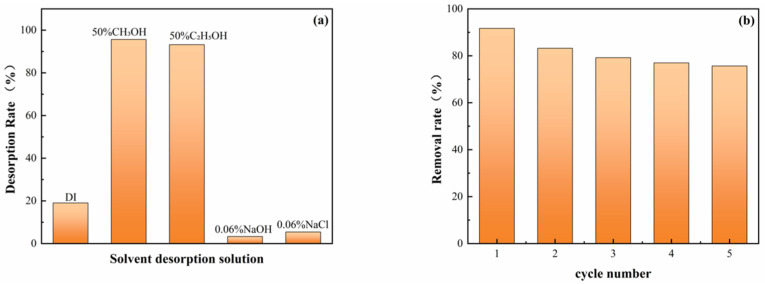
Desorption rate of KBC-900 in different solutions (**a**); PFOA removal efficiency after five adsorption cycles in 50% methanol (**b**).

The recyclability of biochar is one of the key indicators for evaluating its practical application value. As shown in Figure 10, KBC-900 exhibits significant variations in desorption rates across different desorption solvents, with the highest desorption rate of 95.12% achieved in a 50% methanol solution. To further investigate its reusability, KBC-900 underwent five consecutive adsorption cycles using 50% methanol as the regeneration solvent. Figure 10b indicates that the material’s PFOA removal efficiency decreased from 91.68% in the first adsorption cycle to 83.21% in the second cycle. Subsequent cycles showed stable performance without further significant decline, with an adsorption efficiency of 75.69% in the fifth cycle. This phenomenon demonstrates that KBC-900 maintains relatively stable adsorption performance throughout multiple regeneration cycles, exhibiting excellent potential for repeated use.

## 3. Materials and Methods

### 3.1. Materials, Reagents, and Instruments

Waste bamboo powder was obtained from the Chengdu market. After washing and drying in a constant-temperature drying oven at 105 °C, it was pulverized and ground through a 100-mesh sieve for later use. Perfluorooctanoic acid (PFOA, sodium salt) was purchased from Shanghai McLean Company in Shanghai, China. Methanol (AR), potassium hydroxide (KOH, GR, 85%), hydrochloric acid (HCl, 36%), and ethanol (AR) were all supplied by Aladdin Company, Shanghai, China.

Instruments: Tube Furnace, Hefei Kejing, OTF-1200X, Hefei, China; Scanning Electron Microscope (with aSTEM detector), Carl Zeiss, aSTEM detector, Oberkochen, Germany; Surface Area Analyzer, MicrotracBEL, Belsorp max, Osaka, Japan; FTIR Spectrometer, Shimadzu, IRAffinity-1, Kyoto, Japan; Raman Spectrometer, Renishaw, inVia Qontor, Wotton-under-Edge, UK; X-ray Diffractometer, Bruker, D8 VENTURE, Karlsruhe, Germany; Nanoparticle Size & Zeta Potential Analyzer, Malvern Panalytical, Zetasizer Nano ZS90, Malvern, UK.

### 3.2. Preparation of Adsorbents

**Figure 11:**
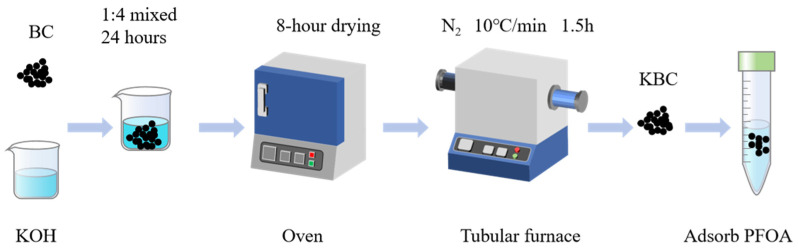
Schematic Diagram of the KBC Series Biochar Preparation Process.

Reserve bamboo powder was placed in a tube furnace and pyrolyzed at 500 °C for 1.5 h under N_2_ atmosphere with a heating rate of 10 °C/min. After removal and cooling, the product was designated BC. BC was mixed with solid KOH (AR) at a mass ratio of 1:4 in a beaker. Slowly add 100 mL water, then place the mixture in a heat-collecting constant-temperature magnetic stirrer with water temperature at 30 °C. Stirred at 500 rpm for 24 h. The mixture was then removed and dried. The dried material was placed in a tube furnace and pyrolyzed under a N_2_ atmosphere at a heating rate of 10 °C/min to activation temperatures of 700 °C, 800 °C, and 900 °C for 1.5 h each. After removal, wash multiple times with 1 M HCl solution, followed by deionized water until the wash water pH is neutral. Dry in an oven to constant weight, designated as KBC-700, KBC-800, and KBC-900 respectively. The preparation process is shown in Figure 11.

### 3.3. Characterization of Adsorbents

Observe the surface morphology of the sample using SEM, determine the adsorption–desorption isotherm of the sample using a specific surface analyzer, calculate the total specific surface area of the sample via the BET equation, and compute the pore size distribution of micropores (<2 nm) and mesopores (2–50 nm) using the HK and BJH methods, respectively. Measure the total pore volume and pore size distribution curve based on the NLDFT model; FT-IR analysis was performed to characterize functional groups within the sample, with a scanning range of 500–4000 cm^−1^. Raman spectroscopy was employed to assess the degree of graphitization defects in the material. X-ray diffraction (XRD) analysis was conducted at angles ranging from 5° to 90°. The zeta potential of the samples was measured using a zeta potential analyzer. Samples were ground to a particle size <50 μm, prepared as a 0.05 g/L solution, and sonicated for 1 h to ensure uniform suspension in ultrapure water. The pH of the solution was adjusted to 2.0–10.0 using NaOH or HCl solutions, and the zeta potential was measured at 25 °C.

### 3.4. Adsorption Experiment

#### 3.4.1. Biochar Dosage and pH Value Effects Experiment

All batch adsorption experiments were conducted in a water bath thermostatic shaker at 250 rpm for 48 h. In the biochar dosage study, the initial PFOA solution concentration was 150 mg/L. Twenty milliliters of solution were transferred to 50 mL PP centrifuge tubes. KBC-900 (added at doses ranging from 5 to 25 mg) was added to the tubes, which were then placed in a water bath shaking incubator set at 298 K. For the pH influence study, the initial solution concentration was 150 mg/L. Transfer 20 mL of solution into a 50 mL PP centrifuge tube. Adjust the pH to 3–10 using 0.1 M hydrochloric acid and 0.1 M sodium hydroxide. Add 10 mg of KBC-900 biochar dispersed in solutions at different pH values and set the temperature to 298 K.

#### 3.4.2. Adsorption Kinetics

In adsorption kinetics studies, 40 mL of PFOA solution (100 mg/L, pH = 3) was added to a 50 mL centrifuge tube. After adding 10 mg of adsorbent, the tube was placed in a constant-temperature shaking incubator at 298 K. At specified time points (0, 0.08, 0.25, 0.5, 1, 2, 4, 6, 8, 12, 24, 48 h), samples were collected by transferring an appropriate volume of solution.

All experiments were conducted with three parallel controls, including a blank control without adsorbent addition. The adsorption of PFOA by the samples was analyzed using the pseudo-first-order kinetic model (Equation (1)) and pseudo-zero-order kinetic model (Equation (2)) to investigate the adsorption mechanism.

(1) $$q_{t}=q_{e}(1-e^{{-K}_{1}t})$$

(2) $$q_{t}=\frac{q_{e}^{2}K_{2}t}{1+q_{e}K_{2}t}$$

In the equation: q_e_—equilibrium adsorption capacity of biochar, mg g^−1^; q_t_—adsorption capacity of biochar at time t, mg g^−1^; K_1_—quasi-first-order kinetic constant, h^−1^; t—adsorption time, h; K_2_—quasi-second-order kinetic constant, g/(mg h).

#### 3.4.3. Adsorption Isotherms

Add 10 mg of adsorbent to a 50 mL centrifuge tube, then add 40 mL of PFOA solution (concentration ranging from 5 to 120 mg/L). Incubate the mixture in a shaking incubator at 250 rpm for 48 h at three temperatures, 298 K (25 °C), 308 K (35 °C) and 318 K (45 °C), then take samples for measurement.

Adsorption isotherms were plotted using the initial mass concentration (C_0_), equilibrium mass concentration (C_e_), and equilibrium adsorption capacity (q_e_) of the adsorbate solution. The Langmuir (Equation (3)) and Freundlich (Equation (4)) models were employed to analyze the isotherm parameters for PFOA adsorption by biochar.

(3) $$q_{e}=\frac{K_{L}q_{m}C_{e}}{1+K_{L}C_{e}}$$

(4) $$q_{t}=K_{F}C_{e}^{1/n}$$

In the equation: q_e_—equilibrium adsorption capacity, mg g^−1^; q_m_—maximum adsorption capacity, mg g^−1^; C_e_—mass concentration at solution equilibrium, mg/L; n—constant related to adsorption strength; K_L_—Langmuir equilibrium adsorption constant; K_F_—Freundlich adsorption capacity constant.

The combined Sips model, incorporating both Langmuir and Freundlich models, was also employed for analysis.

(5) $$q_{e}=\frac{q_{\mathrm{ms}}K_{s}C_{e}^{\beta }}{1+K_{s}C_{e}^{\beta }}$$

In the equation: q_ms_—maximum adsorption capacity, mg g^−1^; K_S_—Sips constant L/mg; β—Sips index describing the uniformity or non-uniformity of the adsorption process.

#### 3.4.4. Adsorption Thermodynamics

The effect of temperature on the adsorption of PFOA by the adsorbent was investigated, with the calculation formula as follows:

(6) $$K_{D}=\frac{q_{e}}{C_{e}}$$

(7) $$\Delta K_{0}=-{\mathrm{RTlnK}}_{D}$$

(8) $$\Delta G_{0}=\Delta H-T\Delta S$$

(9) $${\mathrm{lnK}}_{D}=\frac{\Delta S_{0}}{R}-\frac{\Delta H_{0}}{\mathrm{RT}}$$

In the equation, K_D_ represents the adsorption equilibrium constant (L/g); R denotes the gas constant (8.314 J/(mol·K)); T is the temperature in kelvins (K); ΔG_0_, ΔH_0_, and ΔS_0_ respectively represent the Gibbs free energy (kJ/mol), enthalpy (kJ/mol), and entropy (J/(mol·K)).

#### 3.4.5. Recycling and Practical Application Experiments

The experimental application utilized pure water, tap water, and river water to prepare simulated wastewater. The river water was collected from Yingming Lake on the Chengdu University campus. Use a 0.45 µm needle filter to remove suspended solids from river water, simulate a PFOA concentration of 10 μg/L in wastewater, and maintain other experimental conditions consistent with the adsorption kinetics experiment.

Place saturated spent biochar into 100 mL of different solutions (deionized water, 50% methanol solution, 50% ethanol, 0.06% NaOH, 0.06% NaCl) for desorption. Desorption was conducted in an orbital shaker at 250 rpm for 24 h. After removal, the biochar was rinsed with pure water and dried in a 105 °C oven for 6 h. Under identical adsorption conditions, PFOA adsorption was performed again. The optimal desorption solution was selected for five adsorption–desorption cycles, and for 24 h. After removal, the biochar was rinsed with pure water and dried in a 105 °C oven for 6 h. Under identical adsorption conditions, PFOA adsorption was performed again. The optimal desorption solution was selected for five cycles of adsorption, desorption, and re-adsorption experiments to evaluate the reusability of the biochar.

### 3.5. Data Statistics and Analysis

All data are presented as mean ± standard deviation, with three parallel experiments per group. PFOA concentrations in samples were measured using high-performance liquid chromatography–mass spectrometry (HPLC-MS). The amount of PFOA adsorbed by biochar was calculated according to Equation (10).

(10) $$q_{t}=\frac{(C_{0}-C_{t})V}{m}$$

In the equation, qt represents the adsorption amount at time t, in mg g^−1^; C_0_ denotes the initial solution concentration, in mg/L; C_t_ indicates the concentration at time t, in mg/L; V signifies the solution volume, in L; and m represents the biochar dosage, in g.

## 4. Conclusions

This study demonstrates that bamboo powder biochar (KBC-900), prepared via KOH activation and high-temperature pyrolysis (900 °C), exhibits outstanding adsorption performance for PFOA in water due to its highly developed pore structure (specific surface area of 2924.7 m^2^ g^−1^), highly aromatic hydrophobic surface, and abundant carbon defects. The adsorption process conforms to pseudo-first-order and pseudo-second-order kinetics as well as the Freundlich isotherm model. The theoretical maximum adsorption capacity of PFOA on KBC-900 is 487.89 mg g^−1^. The adsorption of PFOA onto the modified biochar is primarily chemisorption, occurring as a multi-layer adsorption process on a heterogeneous surface. This process is spontaneous and exothermic. Electrostatic attraction, hydrophobic interactions, and π-π interactions collectively constitute the primary adsorption mechanisms. Furthermore, the material maintained relatively stable removal capacity after five regeneration cycles in 50% methanol, demonstrating excellent regenerative performance and potential for application in diverse water bodies. This provides an efficient and reliable solution for removing PFOA from water.

## Figures and Tables

**Table 1:** Specific surface area, mesoporous and micropore area, volume and total pore volume, average pore diameter of samples.

Sample	SBET (m^2^ g^−1^)	Smeso (m^2^ g^−1^)	Smicro (m^2^ g^−1^)	Vmicro (cm^3^ g^−1^)	Vmeso (cm^3^ g^−1^)	Total Pore Volume (cm^3^ g^−1^)	Average Pore Diameter (nm)
KBC-700	2398.6	689.7	1708.9	0.8420	0.4680	1.3100	2.3194
KBC-800	2896.7	835.4	2061.3	0.9780	0.5210	1.4990	2.4294
KBC-900	2924.7	1090.9	1833.8	1.0258	0.5540	1.5798	2.7626

**Table 2:** Adsorption kinetic parameters of PFOA by KBC-series biochar.

Kinetic Models	Parameters	KBC-700	KBC-800	KBC-900
Pseudo-first-order	q_e_ (mg g^−1^)	299.41	329.08	362.31
	K_1_ (h^−1^)	0.535	0615	0.695
	R^2^	0.989	0.986	0.991
Pseudo-second-order	q_e_ (mg g^−1^)	305.74	336.35	369.49
	K_2_ (g mg^−1^ h^−1^)	0.00289	0.00262	0.0024
	R^2^	0.995	0.995	0.995

**Table 3:** Fitting parameters of Langmuir, Freundlich, and Sips models at different temperatures.

Sample	T(K)	Langmuir			Freundlich			Sips			
		q_m_ (mg g^−1^)	K_L_ (L/mg)	R^2^	K_F_ (L/mg)	1/n	R^2^	q_ms_ (mg/g)	K_S_ (L/mg)	1/β	R^2^
KBC- 700	298	419.79	0.354	0.984	113.053	0.415	0.988	426.5	0.308	0.721	0.998
	308	390.60	0.301	0.980	99.173	0.404	0.982	396.8	0.277	0.421	0.997
	318	362.06	0.274	0.974	89.775	0.391	0.992	368.2	0.251	0.224	0.997
KBC- 800	298	456.04	0.668	0.983	159.511	0.414	0.987	462.3	0.972	0.561	0.997
	308	445.65	0.522	0.987	140.626	0.416	0.987	451.7	0.750	0.421	0.998
	318	433.46	0.438	0.985	127.110	0.415	0.986	439.6	0.590	0.527	0.998
KBC- 900	298	487.89	1.389	0.982	233.053	0.422	0.988	494.2	1.233	0.311	0.997
	308	476.73	1.077	0.985	203.689	0.417	0.987	483.1	1.113	0.421	0.997
	318	466.86	0.888	0.983	183.234	0.414	0.986	473.3	1.012	0.459	0.997

**Table 4:** Studies of PFOA adsorption using activated biochar.

Adsorbents	Experimental Conditions	Equilibrium Time (h)	PFOA Maximum Adsorption Capacity (mg g^−1^)	Adsorption Kinetics	Adsorption Isotherm	Ref.
Modified bamboo biochar	0.1 g·L^−1^, 81 mg/L PFOA, pH = 5	24 h	476	Pseudo-second-order	Freundlich	[60]
H3PO4 and KOH-modified grape leaf biochar	50 mg, 30 mL of 1 mg/L PFOA, pH = 4	1 h	78.90	-	Langmuir	[61]
			57.9			
Activated maize tassel	2.0 g·L^−1^, 100 mg/L PFOA, pH = 7	1 h	380.32	Pseudo-second-order	Freundlich	[62]
Magnetic carbide fiber	0.5 g·L^−1^, 250 mg/L PFOA, pH = 3	1 h	204.7	Pseudo-second-order	Langmuir	[63]
Acid-modified biochar	1.0 g·L^−1^, 100 mg/L PFOA, pH = 7	48 h	45.88	Pseudo-second-order	Dubinin—Radushkevich and Sips	[64]
Maize straw	1.25 g·L^−1^, 50 mg/L PFOA, pH = 7	48 h	38.62	-	Langmuir	[65]
Polyaniline-modified bamboo biochar	0.12 g·L^−1^, 100 mg/L PFOA, pH = 3	12 h	264.6	Pseudo-second-order	Langmuir	[66]
Alkali-modified biochar	0.25 g·L^−1^, 120 mg/L PFOA, pH = 3	12 h	487.89	Pseudo-first and second-order	Freundlich	Present study

**Table 5:** Thermodynamic parameters for PFOA adsorption by KBC-700, KBC-800, and KBC-900 at different temperatures.

Sample	T(K)	ΔG_0_ (kJ/mol)	ΔH_0_ (kJ/mol)	ΔS_0_ (J/(mol·K))	R^2^
KBC-700	298	−13.34	−20.59	−24.38	0.989
	308	−13.04			
	318	−12.86			
KBC-800	298	−15.31	−21.33	−20.24	0.987
	308	−15.07			
	318	−14.92			
KBC-900	298	−17.36	−20.27	−9.83	0.989
	308	−17.21			
	318	−17.17			

## Data Availability

The relevant data pertaining to the findings of this study may be obtained from the corresponding author upon request.
